# Structural validation of oral mucosal tissue using optical coherence tomography

**DOI:** 10.1186/1758-3284-4-29

**Published:** 2012-06-06

**Authors:** Zaid Hamdoon, Waseem Jerjes, Raed Al-Delayme, Gordon McKenzie, Amrita Jay, Colin Hopper

**Affiliations:** 1 Department of Oral & Maxillofacial Surgery, Dental School, Mosul, Iraq; 2 Unit of Oral & Maxillofacial Surgery, UCL Eastman Dental Institute, London, United Kingdom; 3 Department of Surgery, Al-Yarmouk University College, Baghdad, Iraq; 4 UCLH Head and Neck Unit, London, United Kingdom; 5 UCL Department of Surgery, University College London Medical School, London, United Kingdom; 6 Leeds Institute of Molecular Medicine, University of Leeds, Leeds, UK

## Abstract

**Background:**

Optical coherence tomography (OCT) is a non-invasive optical technology using near-infrared light to produce cross-sectional tissue images with lateral resolution.

**Objectives:**

The overall aims of this study was to generate a bank of normative and pathological OCT data of the oral tissues to allow identification of cellular structures of normal and pathological processes with the aim to create a diagnostic algorithm which can be used in the early detection of oral disorders.

**Material and methods:**

Seventy-three patients with 78 suspicious oral lesions were referred for further management to the UCLH Head and Neck Centre, London. The entire cohort had their lesions surgically biopsied (incisional or excisional). The immediate *ex vivo* phase involved scanning the specimens using optical coherence tomography. The specimens were then processed by a histopathologist.

Five tissue structures were evaluated as part of this study, including: keratin cell layer, epithelial layer, basement membrane, lamina propria and other microanatomical structures. Two independent assessors (clinician and pathologist trained to use OCT) assessed the OCT images and were asked to comment on the cellular structures and changes involving the five tissue structures in non-blind fashion.

**Results:**

Correct identification of the keratin cell layer and its structural changes was achieved in 87% of the cohort; for the epithelial layer it reached 93.5%, and 94% for the basement membrane. Microanatomical structures identification was 64% for blood vessels, 58% for salivary gland ducts and 89% for rete pegs. The agreement was “good” between the clinician and the pathologist.

OCT was able to differential normal from pathological tissue and pathological tissue of different entities in this immediate *ex vivo* study. Unfortunately, OCT provided inadequate cellular and subcellular information to enable the grading of oral premalignant disorders.

**Conclusion:**

This study enabled the creation of OCT bank of normal and pathological oral tissues. The pathological changes identified using OCT enabled differentiation between normal and pathological tissues, and identification of different tissue pathologies.

Further studies are required to assess the accuracy of OCT in identification of various pathological processes involving the oral tissues.

## Introduction

Over the past few years, there have been very successful attempts in the diagnosis of oral tissue pathologies using optical biopsy or optical diagnostics. In theory, a beam of light fired into tissue should provide an optical signature of that tissue highlighting the abnormal characteristics on cellular and subcellular levels. The mechanism of the optical diagnostics technologies varies but they are all non-invasive with the aim to provide *in vivo*, real time and cost effective diagnosis. The successful application of this technology would reduce the workload on pathology units and reduce the time the anxious patient has to wait for a diagnosis [1,2].

When it comes to oral tissues, the aim of these technologies is to identify premalignant disorders and early cancer. Oral cancer is the sixth most common cancer worldwide. It represents about 2% of the cancers in the UK with incidence of 5.9/100.000 and prevalence of 1 in 1000. Unfortunately, with all advances in surgery, medical and radiation oncology, the 5-year survival continues to be slightly above 50%. The single greatest determinant of long-term patient survival remains early detection and intervention [1,2].

The most commonly used optical diagnostic technologies include elastic scattering spectroscopy (identification of cellular and subcellular changes), Raman spectroscopy (identification of molecular vibration), microendoscopy (identification of surface morphology changes) and fluorescence spectroscopy (identification of biochemical changes in tissue). The results from the immediate *ex vivo* and the *in vivo* studies, where applicable, were very promising [2].

Optical coherence tomography (OCT), another technology which was first applied in 1991 by Huang et al., is a non-invasive interferometric (superimposing or interfering waves) tomographic imaging modality which allows millimeter penetration with micrometer-scale axial and lateral resolution and provides morphological information similar to pathology [3].

For OCT to become clinically interpretable and relevant, the structures visualized must be correlated with the corresponding tissue microstructures. To date, the interpretation of OCT images has been largely intuitive and empiric.

We aimed in this immediate *ex vivo* study to generate a bank of normative and pathological OCT data of the oral tissues to allow identification of cellular structures of normal and pathological processes and compare it to “gold standard” histopathology with the aim to create a diagnostic algorithm which can be used in the early detection of oral disorders.

## Material and methods

This immediate *ex vivo* study involved 73 patients (29 males and 44 females), with 78 suspicious oral lesions. The mean age was 50 years (range 32–68 years). The patients were referred to the UCLH Head & Neck Centre, London, for further management of their oral lesions.

The study protocol was approved by the Moorfields and Whittington Local Research Ethics Committee. The protocol was devised in cooperation with the Departments of Pathology at University College London and Imperial College.

Informed consent was obtained from each patient explaining the nature of the study. Exclusion criteria involved patients under 18 years of age and those with previous history of cancer of the oral cavity and the oropharyngeal/laryngeal regions.

In this study, we used swept-source frequency-domain optical coherence tomography microscope (Michelson Diagnostics EX1301 OCT Microscope V1.0); the components of which were discussed in a previous study [1-3]. The light source used is a Santec HSL-2000, with an imaging wavelength of 1310 nm, axial optical resolution of <10 μm, and lateral optical resolution of <10 μm. The system provides an image resolution of 5.3μmpixel with a maximum image width of 6 mm, a sub-surface imaging depth of 1.5 mm, and a focal depth of 1 mm. Samples can be manipulated to see the full quality results on the screen instantly, with an image capture time of <100 ms and refresh rate of >1 Hz.

The multi-beam swept source OCT EX1301 (Michelson Diagnostics Ltd., Orpington, UK) utilizes a novel optical set-up involving multiple optical channels which does not suffer from loss of sensitivity or other serious drawbacks. The idea is to partition the depth of field into sub-fields and to provide a separately focused beam for each sub-field.

The laser beam in the SS-OCT EX1301 is split into 5 ‘beam-lets’ using an etalon-type ‘rattle plate’ prior to the interferometer beam splitter. Four of these beams are used to scan the specimens and are relayed back to an array of photodiodes where they interfere with four reference beams in the conventional manner. The fifth beam is imaged onto a 5^th^ photodiode to generate a balance signal.

The entire cohort has had their lesions surgically biopsied (incisional or excisional). The specimens were kept in normal saline before being transferred to be scanned. The OCT instrument captured b-mode scans of the tissue. The specimens were then processed by a histopathologist.

Prior to this, digital pictures and diagrams were produced to ensure that the histopathologist would be able to identify the scanned planes accurately and provide an exact histopathological image. Our co-registration method was enhanced by using dyes and sutures for better orientation.

A histopathological diagnosis was then achieved after several steps including tissue sample embedding in paraffin wax, staining with haematoxylin and eosin (H&E), and examination by light microscopy. Close attention was paid to tissue shrinkage in formalin when comparing microanatomical structures of immediate *ex vivo* OCT images and paraffin wax slides.

Five tissue structures were evaluated as part of this study, including: keratin cell layer, epithelial layer, basement membrane, lamina propria and other microanatomical structures. Two independent assessors (clinician and pathologist trained to use OCT) assessed the OCT images and were asked to comment on cellular structures and changes involving the five tissue structures in non-blind fashion.

Seven variables were studied on the OCT images to identify normal oral mucosa microanatomical structures and architectural changes in these areas caused by the pathological processes. This included visibility of keratin layer, epithelial layer and lamina propria, identification of the basement membrane and identification of blood vessels, minor salivary gland ducts, rete pegs and taste bud papilla (where applicable). Structural changes of keratin layer, epithelial layer and lamina propria were also examined and highlighted.

OCT measurements were taken from the edges of the macroscopically normal oral mucosa of the surgical biopsy (assumed to be normative data); while pathological data were acquired from the centre of the lesion.

### Statistical analysis

All data were entered and stored in a computerized database (Microsoft Excel 2010). The statistical analysis was performed by using the statistical software package SPSS 13.0 (SPSS, Chicago, Ill).

## Results

Clinical examination of the suspicious oral lesions was reported and is highlighted in Table 1. The lesions were mainly identified in the tongue, buccal mucosa and floor of mouth. Histopathological diagnosis revealed that dysplasia (oral potentially malignant disorder) was identified in 30 patients, carcinoma *in situ* in 4 patients and squamous cell carcinoma (SCC) in 25 patients (Table 1).

**Table 1:** Characteristics of imaged lesions demographic location

	**No. (%)**		**No. (%)**
**Gender**		**Clinical features**	
Male	44 (60.2)	Papule	26 (33.3%)
Female	29 (39.8)	Plague	22 (28.2)
		Ulcer	18 (23%)
**Location**		Others	12 (15.3)
Tongue	30 (38.4)		
Buccal mucosa	21 (26.9)	**Symptoms**	
Floor of mouth	13 (16.6)	Oral discomfort	29 (39.7)
Hard palate	8 (10.2)	Symptomless	20 (27.3)
Soft palate	6 (7.6)	Pain	13 (17.8)
		Bleeding	11 (15)
**Colour**			
Leukoplakia	26 (33.3)	**Histologic diagnosis**	
Speckled leukoplakia	24 (30)	Dysplasia	30 (38.4)
Erythroplakia	10 (12.8)	Carcinoma in situ	4 (5.1)
Bluish	10 (12.8)	Invasive carcinoma	25 (32)
Normal	8 (10.2)	Frictional keratosis	5 (6.5)
		Other Benign lesion	14 (18)

### OCT and histopathology correlation

**Figure 1:**
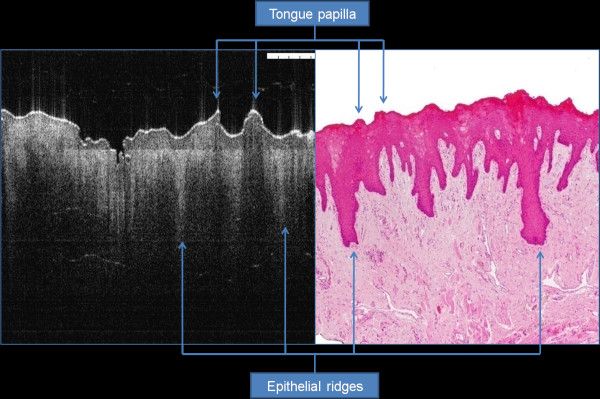
H&E and OCT corresponding images of tongue biopsy showing prominent epithelium ridges and tongue papilla.

**Figure 2:**
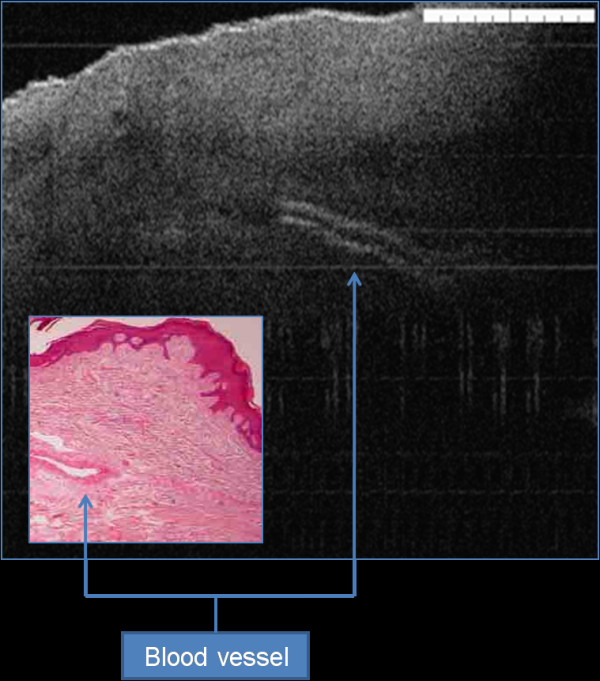
Prominent blood vessel appears as two lines with hyper-ecoic signal and central hypo-ecoic shadow.

**Figure 3:**
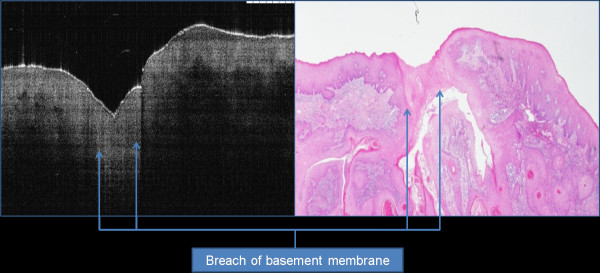
Focal invasive carcinoma of buccal mucosa with localized breach of basement membrane and thin or no keratin cell layer.

**Figure 4:**
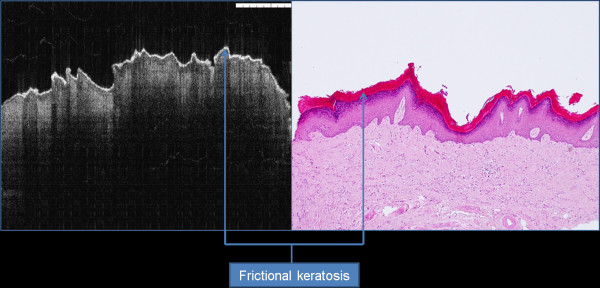
Frictional keratosis showing hyper-reflective OCT signal.

**Figure 5:**
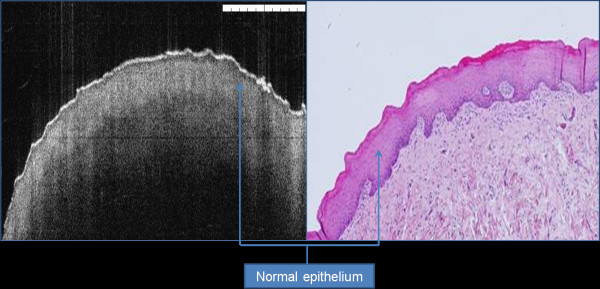
**Normal epithelium showing normal reflective keratin cell layer, basement membrane and homogenous lamina propria.** The area between the basement membrane and upper most layers represent oral epithelium.

OCT imaging showed distinct zones of normal and altered architectural changes. Basic histological layers (keratin cell layer, epithelium, lamina propria) and microanatomical structures (i.e. blood vessels, tongue papillae, and glandular ducts) were identified in most of the images (Figures 1, 2, 3, 4, 5). The basement membrane was clearly identified in many specimens.

Structural identification and validation with histopathology was variable. This was achieved in 97% when assessing the basement membrane, 93.5% in identifying epithelial layer and its changes and 94% in assessing keratin cell layer and its changes. Correlation was less achievable when it comes to blood vessels (77%) and salivary gland ducts (60%). Rete ridges were correlated in 89% of the OCT specimens and validated histopathologically (Table 2). For normal resection margins, OCT and histology images were compared and high correlation was achieved (Table 3).

**Table 2:** Common descriptive features between OCT and pathology from oral tissue agreed by two observers

	**No.**	**No.**	**No.**
**Keratin cell layer**	**OCT**	**Pathology**	**Total**
Clearly seen	62	66	128
Not seen	16	12	28
Total	78	78	156
**Basement membrane**	**OCT**	**Pathology**	**Total**
Clearly identified	70	68	138
Not identified	8	10	18
Total	78	78	156
**Blood vessel in the lamina propria**	**OCT**	**Pathology**	**Total**
Clearly seen	17	22	39
Not seen	61	56	117
Total	78	78	156
**Tongue papilla**	**OCT**	**Pathology**	**Total**
Clearly seen	14	16	30
Not seen	16	14	30
Total	30	30	60
**Epithelium boundary**	**OCT**	**Pathology**	**Total**
Clearly seen	69	70	139
Not seen	9	8	17
Total	78	78	156
**Salivary gland duct**	**OCT**	**Pathology**	**Total**
Clearly seen	6	10	16
Not seen	72	68	140
Total	78	78	156
**Rete ridges**	**OCT**	**Pathology**	**Total**
Clearly seen	55	62	117
Not seen	23	16	39
Total	78	78	156

**Table 3:** Common descriptive features between OCT and histology from normal resection oral tissue agreed by two observers.

	**No.**	**No.**	**No.**
**Keratin cell layer**	**OCT**	**Histology**	**Total**
Clearly seen	30	30	60
Not seen	0	0	0
Total	30	30	60
**Basement membrane**	**OCT**	**Histology**	**Total**
Clearly identified	30	30	60
Not identified	0	0	0
Total	30	30	60
**Blood vessel in the lamina properia**	**OCT**	**Histology**	**Total**
Clearly seen	12	15	27
Not seen	18	15	33
Total	30	30	60
**Tongue papilla**	**OCT**	**Histology**	**Total**
Clearly seen	10	10	20
Not seen	20	20	40
Total	30	30	60
**Epithelium boundary**	**OCT**	**Histology**	**Total**
Clearly seen	30	30	60
Not seen	0	0	0
Total	30	30	60
**Salivary gland duct**	**OCT**	**Histology**	**Total**
Clearly seen	6	7	13
Not seen	24	23	47
Total	30	30	60
**Rete ridges**	**OCT**	**Histology**	**Total**
Clearly seen	22	25	47
Not seen	8	5	13
Total	30	30	60

### Description of keratin layer

In normal keratinised mucosa, the keratin layer appears as thin bright line on the most upper part of epithelium. This layer is absent in non-keratinised epithelium. In frictional keratosis, this layer shows hyper-reflection features with moderate thickening. Similarly, other benign oral conditions show high backscattering signals similar to reactive keratosis (Figure 4).

Dysplasia cases (mild, moderate) mainly demonstrate hyper-signal, however, severe dysplasia and carcinoma *in situ* have hypo-reflective keratin layer due to the disorganised tissue differentiation. Invasive carcinoma can either have hypo-reflective keratin layer or no layer due to structural damage from ulceration.

### Description of epithelial layer

Epithelial layer in normal mucosa has relatively lower signal intensity than keratin cell layer and lamina propria. This layer has homogenous structure with hard distinction between spinous and granular cell layers (Figure 5). In benign lesions this layer may show slight increase in the thickness mainly when the lesion show tissue hyperplasia.

In dysplastic cases, slight to moderate increase in this layer thickness and is usually associated with architectural changes in severe dysplasia and carcinoma *in situ*. In cases of invasive carcinoma, epithelial layer shows significant increase in the thickness in the areas of focal invasion.

### Description of lamina propria layer

In normal tissue, this layer show noticeable demarcation from the upper epithelium with high signal. Small blood vessels may be seen as poor signal surrounded by two high signal lines (Figure 2).

### Description of basement membrane

The demarcation between two different signal intensity of epithelium and lamina propria represents the basement membrane. This junction may appear as linear of undulated structure due to tissue formalin shrinkage effect. Small projections towards the lamina propria may be seen which represent ret pegs. Intact basement membrane is seen in benign and dysplastic cases, while complete or partial loss (breach) of the basement membrane is usually evident in cases of invasive carcinoma (Figure 3).

### Description of other microanatomical structures

In the normal part of dorsum of tongue biopsy, mushroom or feather-like humps can be identified and represent the fungi-form and filli-form papilla (Figure 1). Minor salivary gland ducts may be seen in some biopsies as tiny tortuous signal free cavities which can be difficult to differentiate from blood vessels. Rete pegs appear as shadow extension from the epithelium holding the same signal intensity.

### Agreement between the clinician and the histopathologist

With regards to hyperkeratosis, agreement was achieved in 100% of the specimens in identifying the basement membrane and epithelial layer and its changes. 100% of the keratin cell layer demonstrated hyper-reflective features. With regards to other benign oral lesions, agreement was achieved in 78% of the specimens in identifying the basement membrane as demarcated structure, 78% in identifying epithelial layer as normal thickness and 92% in keratin cell layer described as normal reflective.

In the oral dysplasia group, agreement was achieved in 90% of the specimens in identifying the basement membrane, 83% in identifying epithelial layer as increased, and 13% in identifying hypo-reflective keratin cell layer. For oral cancer, agreement was achieved in 100% of the specimens in identifying the status of the basement membrane and 100% in identifying thickened epithelial layer (Table 4).

**Table 4:** Descriptive interpretation of OCT image changes. KL: keratin cell layer, EP: epithelium, BM: basement membrane, LP: lamina propria. Increase: ↑, decrease: ↓, no change: ↔

**Pathology entity**	**KL hyper-reflective**	**KL hypo-reflective**	**KL normo-reflective**	**Not applicable**	**EP ↑**	**EP ↓**	**EP ↔**	**EP not identified**	**BM demarcated**	**BM non-demarcated**	**Not applicable**	**LP homogenous**	**LP non-homogenous**
Keratosis (n = 5)	5	0	0	0	1	0	4	0	5	0	0	5	0
Benign oral lesions (n = 14)	2	0	9	3	1	1	11	1	11	2	1	12	2
Oral dysplasia (n = 30)	13	4	10	3	25	0	3	2	27	1	2	26	4
Carcinoma in situ (n = 4)	1	1	2	0	4	0	0	0	4	0	0	4	0
Invasive carcinoma (n = 25)	10	10	5	0	25	0	0	0	1	24	0	0	25
Normal margin (n = 30)	0	0	30	0	0	0	30	0	30	0	0	30	0

### Diagnostic criteria

By analysing an OCT image, the normal oral mucosa exhibited three layers. The upper portion of the mucosa (keratin cell layer) appeared distinct and more reflective than the rest of the epithelium. Basement membrane appeared as change of contrast between the hyper-reflective epithelium and hypo-reflective lamina propria. Some epithelium characterized by prominent ret peg ridges which clearly demarcate the intact basement membrane. Microanatomical structures including blood vessels, salivary gland ducts were seen in the third layer, which was the lamina propria.

In the examination of an invasive carcinoma, OCT images showed break down of the basement membrane which correlated well with the histological findings of the same specimen. Cancerous lesions also showed irregular and unclear architecture in the lower lamina propria as non-homogenous structure. The majority of the invasive lesions had hypo-reflective signals for the keratin cell layer, while epithelium thickness increased in all OCT images scanned. Advanced malignant lesions extended in deeper tissue layers were beyond the scope of this technology as OCT cannot penetrate deeper than 2 mm in tissue.

OCT was able to differentiate normal from pathological tissue and pathological tissue of different entities. OCT failed to provide enough cellular and subcellular information for staging of oral premalignant disorders (potentially malignant). Differentiation between normal and pathological tissue was mainly based on the identification of thickened oral epithelium and disorganized keratin layer and subepithelial structures.

Differentiation between invasive carcinoma and other benign entities was accurate based on basement membrane status (intact or breached). The epithelium in the early invasive carcinoma is highly variable in thickness, with areas of invasion into the subepithelial layers with invisible basement membrane. However, the OCT image of a dysplastic lesion showed epithelial thickening without frank breach of the basement membrane; this was sometimes difficult to be differentiated from some benign lesions. Thickness of the epithelium for two lesions with carcinoma *in situ* has higher value for benign lesions removed from the same anatomic area.

## Discussion

The term “optical biopsy” refers to the method that uses the properties of light to enable the operator to make a “real time” diagnosis. However, the term optical biopsy is misnomer as biopsy mean surgical tissue removal. Optical diagnostics seems a correct terminology for these techniques [1-3].

Although OCT can accurately evaluate frank cancer, oral premalignant disorders can be difficult to stage. Dysplasia is identified by cytological and architectural changes. The former including nuclear size and shape, nuclear/cytoplasmic ratio and nuclear stratification, are beyond the resolution of OCT. Visualization of subcellular features, such as nuclei (size, number, and chromatin content) and organelles was impossible in the current premises.

Several studies have sought to investigate the diagnostic utility of OCT to detect and diagnose oral and oropharyngeal/laryngeal pre-malignancy and malignancy [4-6]. No study adhered to solid diagnostic criteria to reach the diagnosis mainly for oral premalignant disorders.

In one study, involving 50 patients with suspicious lesions including oral leukoplakia or erythroplakia, the effectiveness of OCT was evaluated for detecting oral premalignant disorders and malignancy [7]. OCT images of the dysplastic lesions revealed visible epithelial thickening, loss of epithelial stratification and epithelial down growth. This criterion is not adamant enough to draw firm conclusion and to grade oral premalignant disorders.

Criteria for oral cancer diagnosis was established using OCT images by the absence or disruption of the basement membrane and the epithelial layer that was highly variable in thickness, with areas of erosion and extensive epithelial down growth and invasion into the subepithelial layers.

In another study [8], 97 patients were subjected to OCT imaging to detect neoplasia in the oral cavity. The results revealed that the main diagnostic criterion for high-grade dysplasia and carcinoma *in situ* was the lack of a layered structural pattern. Diagnosis based on this criterion for dysplastic⁄malignant vs. benign ⁄ reactive conditions was achieved. In a similar study conducted by our group, OCT images of suspicious oral lesions obtained in *ex vivo* form. It was found that OCT was able to distinguish various layers of oral mucosa. In addition segregating benign from malignant lesion was easily feasible [9].

One limitation of the current study is the co-registration of images with histopathology. The method used in this study provides an accurate registration of OCT and pathology. However, the exact control of the histopathological plane may be difficult due to the formalin shrinkage effect and the processing artifacts in histology.

A second limitation is the relatively small sample size which prevents us from determining the sensitivity and specificity as well as the accuracy for assessment of oral diseases. This study has been sufficient to suggest possible applications of this technique in the oral cavity. Also, further advances and modification of OCT probe technology will be needed to make OCT suitable for routine *in vivo* clinical use.

A third limitation is that the image quality was affected due to lack of tissue perfusion (*ex vivo* nature of the study). The use of high resolution *in vivo* optical imaging may offer a clinically useful adjunct to standard histopathological techniques.

Ridgway et al. examined the mucosa of the oral cavity and the oropharynx using OCT in 41 patients during operative endoscopy [10]. OCT imaging was combined with endoscopic photography for correlation of gross and histological images. They concluded that OCT images of the oral cavity and oropharynx provided microanatomical information about the epithelium, basement membrane, and supporting lamina propria of the mucosa, and showed distinct zones of normal, altered, and ablated tissue microstructures for each pathological process studied.

Building on the results from this study, the diagnostic criteria should be applied through a blind study to judge robustly the sensitivity and accuracy for diagnosis of oral lesions before considering these criteria as gold standard for future work.

## Conclusion

This study validates the use of OCT in identifying structural changes in healthy and pathological oral mucosa. Further studies to prove its *in vivo* usefulness are required.

## Competing interests

Mr Colin Hopper is an Advisory Board Member at Michelson Diagnostics, Kent, UK. Dr Gordon McKenzie is a Medical Applications Director at Michelson Diagnostics, Kent, UK.

## Authors’ contribution

**ZH, WJ, AJ, CH** designed and performed the study, carried out the literature research and manuscript preparation. **ZH, WJ, RA, GM, AJ, CH** were responsible for critical revision of scientific content and manuscript review. All authors approved the final version of the manuscript.
